# Defensive Properties of Mucin Glycoproteins during Respiratory Infections—Relevance for SARS-CoV-2

**DOI:** 10.1128/mBio.02374-20

**Published:** 2020-11-12

**Authors:** Maitrayee Chatterjee, Jos P. M. van Putten, Karin Strijbis

**Affiliations:** a Department Biomolecular Health Sciences, Division Infectious Diseases and Immunology, Faculty of Veterinary Medicine, Utrecht University, Utrecht, The Netherlands; University of Texas Health Science Center at Houston

**Keywords:** mucosal barrier, mucin, MUC1, O-linked glycans, glycosylation, respiratory pathogens, mucin-microbe interactions, SARS-CoV-2

## Abstract

Mucus plays a pivotal role in protecting the respiratory tract against microbial infections. It acts as a primary contact site to entrap microbes and facilitates their removal from the respiratory tract via the coordinated beating of motile cilia. The major components of airway mucus are heavily *O*-glycosylated mucin glycoproteins, divided into gel-forming mucins and transmembrane mucins. The gel-forming mucins MUC5AC and MUC5B are the primary structural components of airway mucus, and they enable efficient clearance of pathogens by mucociliary clearance. MUC5B is constitutively expressed in the healthy airway, whereas MUC5AC is upregulated in response to inflammatory challenge. MUC1, MUC4, and MUC16 are the three major transmembrane mucins of the respiratory tracts which prevent microbial invasion, can act as releasable decoy receptors, and activate intracellular signal transduction pathways. Pathogens have evolved virulence factors such as adhesins that facilitate interaction with specific mucins and mucin glycans, for example, terminal sialic acids. Mucin expression and glycosylation are dependent on the inflammatory state of the respiratory tract and are directly regulated by proinflammatory cytokines and microbial ligands. Gender and age also impact mucin glycosylation and expression through the female sex hormone estradiol and age-related downregulation of mucin production. Here, we discuss what is currently known about the role of respiratory mucins and their glycans during bacterial and viral infections of the airways and their relevance for the novel coronavirus severe acute respiratory syndrome coronavirus 2 (SARS-CoV-2). Understanding the impact of microbe-mucin interaction in the respiratory tract could inspire the development of novel therapies to boost mucosal defense and combat respiratory infections.

## Abstract (precis)

Mucus plays a pivotal role in protecting the respiratory tract against microbial infections. It acts as a primary contact site to entrap microbes and facilitates their removal from the respiratory tract via the coordinated beating of motile cilia. The major components of airway mucus are heavily *O*-glycosylated mucin glycoproteins, divided into gel-forming mucins and transmembrane mucins. The gel-forming mucins MUC5AC and MUC5B are the primary structural components of airway mucus, and they enable efficient clearance of pathogens by mucociliary clearance.

## INTRODUCTION

## MUCUS: FIRST LINE OF DEFENSE IN THE RESPIRATORY TRACT

**FIG 1:**
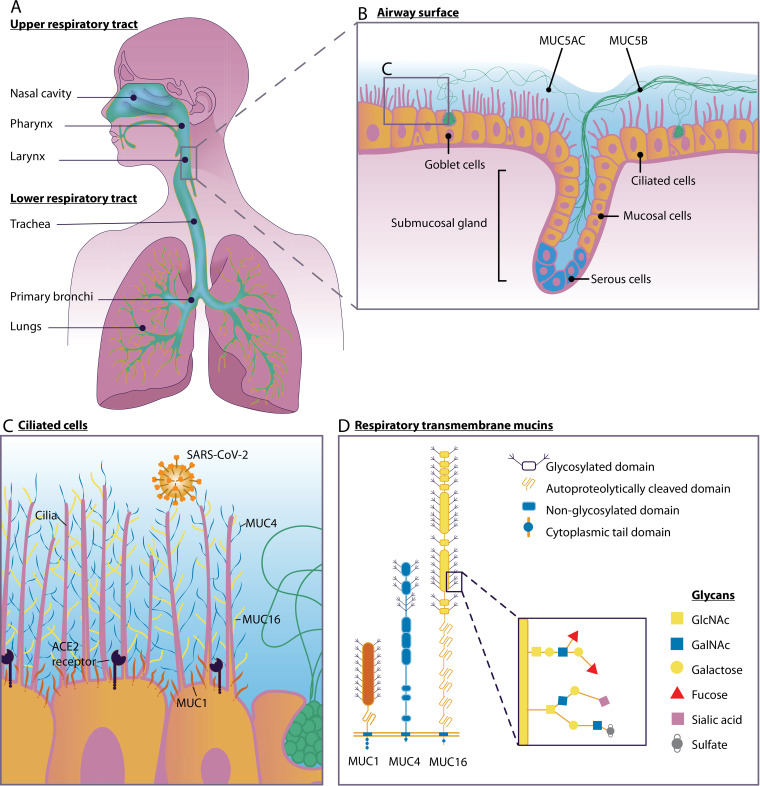
Mucosal defense in the respiratory tract during SARS-CoV-2 infection. (A) Human upper and lower respiratory tracts. (B) Respiratory epithelium with ciliated cells, goblet cells, and a submucosal gland. The soluble mucin MUC5AC is secreted by goblet cells, and the soluble mucin MUC5B is secreted by mucosal cells in the submucosal gland. (C) Ciliated epithelial cells express transmembrane mucins MUC1 (red), MUC4 (blue), and MUC16 (yellow) and the SARS-CoV-2 entry receptor ACE2. (D) Domain structure of transmembrane mucins MUC1, MUC4, and MUC16. Mucin *O*-glycan structures and, specifically, terminal sialic acids play an important role in virus-mucin interactions.

Mucus plays a vital role in protecting the respiratory tract from chemical, microbiological, and physical noxious environmental factors. The respiratory tract can be classified into two zones: the upper respiratory airway and the lower respiratory airway. The upper respiratory tract includes the nose, the pharynx, and the larynx, which are located outside the chest cavity, while the lower respiratory tract comprises the trachea, the lungs, and all segments of the bronchial tree (including the alveoli) (Fig. 1A). The respiratory airways are lined with pseudostratified epithelium and are made up of three major cell types: ciliated cells, nonciliated secretory cells (goblet cells), and basal cells (1). Goblet cells secrete soluble mucus that acts as the first line of defense by protecting underlying epithelial cells from invading pathogens (Fig. 1B). The airway mucus layer is part of the innate defense mechanism and is aimed at trapping and keeping the microbes away from the host epithelial cell surface, a process called mucociliary clearance (MCC). MCC requires the coordinated functions of the secretory cells that release mucus and of the motile cilia on multiciliated cells to control the viscoelasticity and resulting transportability of secreted mucus, respectively (2, 3). Due to the MCC, the mucus layer is continuously renewed. The nasal mucus is replaced every 10 min. whereas mucus in the human lower respiratory tract is transported upward with a velocity of about 100 μm per s, resulting in complete turnover within minutes to hours. This high turnover rate is one of the major protective features of the respiratory mucosa (4). The mucus system captures and removes incoming pathogens, thereby preventing invasion of underlying epithelial cells. In this minireview, we summarize what is known about the respiratory mucosal defense system in the context of bacterial and viral infections and discuss its importance during severe acute respiratory syndrome coronavirus 2 (SARS-CoV-2) infection.

## MUCINS, THE PRINCIPAL COMPONENTS OF MUCUS

The principal components of mucus are high-molecular-weight (HMW) mucin glycoproteins. To date, 22 different human mucins genes have been identified. The major mucins produced in the airways are soluble mucins MUC5AC and MUC5B, which are secreted by goblet cells, and the transmembrane mucins MUC1, MUC4, and MUC16, which are expressed on the apical surface of all epithelial cell types along the different tissues of the respiratory tract (5). The glycan structures on the extracellular domains of transmembrane mucins attract water, thereby forming a fluid layer surrounding the cilia. This layer is called the periciliary layer and is essential for normal ciliary action to remove mucus from the airways. A viscoelastic airway mucus gel is located on top of the periciliary layer that consists of gel-forming mucins which have been secreted by goblet cells (6).

Soluble and transmembrane mucins contain highly glycosylated variable-number tandem repeat (VNTR) regions that are rich in proline, threonine, and serine (PTS) repeats. These amino acid residues are potential *O*-glycosylation sites for attachment of complex carbohydrate structures that contain *N*-acetylgalactosamine (GalNAc), *N*-acetylglucosamine, fucose, galactose, sialic acid (*N*-acetylneuraminic acid), and traces of mannose. The *O*-glycan structures that are attached to mucins constitute up to 80% of the total molecular weight of the final mucin glycoprotein. During mucin synthesis, *O*-glycosylation is initiated in the Golgi apparatus after addition of the primary GalNAc to a serine or threonine residue on the mucin protein backbone, resulting in GalNAc-O-Ser/Thr or Tn antigen structures. This first step in mucin *O*-glycosylation can be catalyzed by up to 20 polypeptide GalNAc transferases (ppGalNAcTs). The next step in mucin *O*-glycosylation is the attachment of core 1, 2, and 3 structures. Core 1, also called T antigen, is synthesized by C1GalT1 and consists of Galβ1-3GalNAc structures. Core 1 can be converted to core 2 by the action of C2GnT1-3 enzymes, resulting in Galβ1-3(GlcNAcβ1-6)GalNAc structures. Core 3 is a GlcNAcβ1-3GalNAc structure that is synthesized from Tn antigen by the β3GnT6 enzyme. Once the core structures are attached, 30 or more distinct glycosyltransferases sequentially add other sugar moieties, resulting in the completed *O*-glycan structure. Sialyltransferases and fucosyltransferases often terminate chain elongation and branching, thereby generating mucins with terminal charged sialic acid and hydrophobic fucose residues that represent the interaction surface that is involved when engaging with microbes (7). Mucin expression and O-glycosylation are altered under inflammatory conditions of the respiratory tract and are directly affected by colonization and invasion by bacteria and viruses.

## SOLUBLE GEL-FORMING MUCINS OF THE RESPIRATORY TRACT

The gel forming mucins MUC5AC and MUC5B are the primary macromolecular components of airway mucus and facilitate airway clearance by mucociliary transport. In healthy individuals, MUC5AC is produced by goblet cells in the nasal mucosa and tracheobronchial surface epithelium of the lower respiratory tract (Fig. 1B) (6). In contrast, MUC5B expression is restricted to the lower respiratory tract. MUC5AC and MUC5B play distinct roles in maintaining a healthy airway epithelium. MUC5B is the primary mucin in healthy human airways and is constitutively expressed. MUC5B is secreted as large linear mucus bundles by the mucous cells in the bronchial submucosal glands, which is achieved by the combination of intracellular packing of the mucin and gland morphology (Fig. 1B) (8). The degrees of sialylation of MUC5B differ between healthy and diseased airways, with the highly charged sialylated glycoform being predominant in healthy airways (9). MUC5AC is produced at much lower levels in healthy airways but is upregulated in response to microbial infection and inflammatory cytokines (10). The protein backbones of both MUC5AC and MUC5B contain multidomain structures with central *O*-glycosylated tandem repeats flanked by D, B, C, and CK domains. The two secreted mucins share common glycans and core structures, but MUC5AC is more extensively fucosylated than MUC5B. These fucose groups impart hydrophobic properties on MUC5AC, resulting in inhibition of MCC and mucus turnover under inflammatory conditions (5).

## EPITHELIAL TRANSMEMBRANE MUCINS OF THE RESPIRATORY TRACT

Transmembrane mucins are long extended molecules that protrude from the apical surface of the epithelium. They consist of a heavily *O*-glycosylated N-terminal extracellular domain, a single transmembrane domain, and a C-terminal cytoplasmic domain. In most transmembrane mucins (except MUC4), the glycosylated extracellular domain is noncovalently associated with the transmembrane domain at the sea urchin sperm protein, enterokinase, and agrin (SEA) domain, which is autoproteolytically cleaved in the endoplasmic reticulum (11). MUC1 and MUC4 are expressed in the upper and lower respiratory tracts, whereas MUC16 is expressed only in the lower respiratory tract (12). In the lung, MUC1 mainly localizes around microvilli and extends at least 100 nm from the cell surface. MUC4, which is about 300 nm in size, and the even larger MUC16 are expressed on the surface of cilia (13) (Fig. 1C). MUC1, MUC4, and (possibly) MUC16 are closely associated with the liquid periciliary layer surrounding the microvilli and cilia. Because of their high levels of sialic acid and sulfate content, they provide a strongly negatively charged milieu around the airway epithelia. This facilitates the formation of mucin rafts of secreted mucins MUC5AC and MUC5B and their continuous removal from the lungs (12). Transmembrane mucins themselves form a barrier that prevents pathogen invasion of the underlying epithelium. In addition, pathogen binding can induce serine and/or tyrosine phosphorylation of the cytoplasmic tail (14). Through the combined functions of the extracellular domain and the cytoplasmic tail, transmembrane mucins sense the external environment and activate intracellular signal transduction pathways. Transmembrane mucin-associated signaling pathways have been implicated in controlling inflammation and immune responses to infectious agents and in maintenance of the mucosal barrier (12, 13). While all transmembrane mucin cytoplasmic tails contain putative phosphorylation sites, the tails are highly diverse between different transmembrane mucins. However, tails of specific transmembrane mucins are conserved across species, suggesting important interspecies mucin-specific signaling functions (14).

An additional mechanism of mucin defense is shedding of the extracellular domain from the cell surface into the lumen. When microbes are bound to transmembrane mucins, mucin shedding can result in release of mucin-bound pathogens from the cell and clearance from the body via the MCC (15). Mucin shedding can take place at the noncovalently attached SEA domain or by proteolytic cleavage by endogenous proteases such as disintegrin and metalloprotease domain containing protein-17 (ADAM17) or membrane type 1 matrix metalloprotease (MMP-MT1) (16). The specific mechanisms of transmembrane mucin shedding in the respiratory tract have not been studied. As a group, the different transmembrane mucins provide important protection against invading pathogens during airway infection at several different levels. Studying the functions of individual transmembrane mucins and their glycosylation during infection could provide a promising direction to develop new strategies to improve mucosal defenses.

## CHANGES IN MUCIN EXPRESSION AND GLYCOSYLATION DURING INFLAMMATION

Under inflammatory conditions, expression of soluble and transmembrane mucins is increased, and their glycosylation is altered to boost mucosal defense. These changes alter the interaction interface. The transmembrane mucin MUC1 is upregulated in the oral mucosal epithelia by proinflammatory cytokines interleukin-6 (IL-6), gamma interferon (IFN-γ), and tumor necrosis factor alpha (TNF-α) (17). Lipopolysaccharide (LPS) of the bacterial pathogens Pseudomonas aeruginosa and Haemophilus influenzae enhances MUC5AC expression and impedes mucin glycosylation and sulfation (18). During pulmonary disease, expression of MUC4 is increased in the presence of the inflammatory protease neutrophil elastase, possibly to activate epithelial repair mechanisms (19). TNF-α also alters the sialylation of mucins in tracheal cells and changes the expression of fucosyltransferases and α2,3-sialyltransferases in normal bronchial mucosal explants (20). Th2 cytokines (IL-4, IL-9, and IL-13) and TNF-α increase sialylation and the negative charge of mucins, thereby contributing to defense mechanisms during epithelial injury. Negatively charged mucin carbohydrates inhibit the effect of cationic inflammatory proteins such as human neutrophil elastase and lysozyme as well as bacterial enzymes such as proteases and elastase and glycosidic enzymes which cleave carbohydrates from the mucin glycoprotein (21, 22). As mentioned above, MUC5B is upregulated under inflammatory conditions (10) and its increased fucosylation could negatively impact MCC and mucus turnover (5). Alterations in expression of fucosyltransferases and their fucosylated glycan products have also been observed in inflammatory diseases of the airway such as asthma and cystic fibrosis (17, 19).

## BACTERIAL INTERACTIONS WITH SOLUBLE MUCINS AND MUCIN GLYCANS

The soluble mucins of the respiratory tract pose a strong barrier that prevents invasion by the majority of bacterial and viral pathogens. However, respiratory pathogens such as P. aeruginosa, Staphylococcus aureus, H. influenzae, Streptococcus pneumoniae, and Streptococcus pyogenes have evolved virulence factors to interact with mucins and mucin glycans during the course of airway infection (23). Experiments with mucin knockout and overexpression mice demonstrated that the soluble mucins play key roles in preventing microbial infection. Mice with knockouts for Muc5b were prone to bacterial infections and displayed a number of severe deficiencies in respiratory function. The lower respiratory tract of *Muc5b* knockout mice contained bacteria (particularly S. aureus) and inflammatory infiltrates and decreased levels of IL-23, a mediator of antimicrobial inflammatory responses, and the *Muc5b* knockout mice showed accumulation of impaired phagocytic macrophages in the lungs (24). S. pneumoniae is a commensal bacterium that resides in the human nasopharynx, typically without causing disease. However, under favorable conditions, S. pneumoniae can migrate from the nasopharynx to other sites of the body such as the lungs and bloodstream, causing pneumonia and sepsis. Pneumolysin, a virulence factor secreted by S. pneumoniae, played an important role in MUC5AC induction via Toll-like receptor 4 (TLR4)-dependent activation of extracellular signal-regulated kinase (ERK) both in human epithelial cells and in mice (25).

Several studies have reported that the terminal sugars of mucin *O*-glycans and, particularly, sialic acids play a key role in interactions with respiratory pathogens. S. pyogenes, the causative agent of acute pharyngitis and impetigo, primarily colonizes the upper respiratory pharyngeal mucosa. Streptococci first need to traverse the mucus layer in order to adhere to the underlying epithelial cells. Streptococcal surface M protein interacts with α2-6-linked sialic acid in bovine soluble mucins. Additionally, it was reported previously that sialic acids on cell surface proteins play a significant role in the binding of streptococci to pharyngeal epithelial cells (26). Sialic acids can also serve as binding receptors for P. aeruginosa (27) and H. influenzae (28). The flagellin of P. aeruginosa preferentially binds mucin sialyl-Lewis^x^ moieties, which contain both sialic acid and fucose monosaccharides. Intriguingly, there is evidence that P. aeruginosa can increase the sialyl-Lewis^X^ content on mucins via its secreted virulence factor pyocyanin, thereby making the host environment more favorable for bacterial attachment to surface mucins and colonization (29). Furthermore, it was reported previously that S. pneumoniae prefers to adhere to oligosaccharide ligands terminating in NeuAcα2-3Galβ1 (or NeuAcα2-6Galβ1) in the human airway (30). Similarly, the surface-associated high-molecular-weight protein 1 (HMW1) adhesin of H. influenzae is mostly dependent on α2-3-linked sialic acid (31). Because interactions between pathogens and mucin glycans are very specific, these processes are attractive targets for novel drug development.

## FUNCTIONS OF TRANSMEMBRANE MUCIN DURING RESPIRATORY INFECTIONS WITH BACTERIAL PATHOGENS

MUC1 is the most extensively studied transmembrane mucin and is expressed in both the nasal cavity and lower respiratory tract. MUC1 extends 100 nm from the plasma membrane, localizes around microvilli, and has been shown to play an important defensive role during infection by various bacterial pathogens. P. aeruginosa can cause acute and chronic respiratory tract infections. Various papers have demonstrated that MUC1 plays an anti-inflammatory role during infection with P. aeruginosa. *In vivo* studies with *Muc1* knockout mice showed increased inflammation compared to wild-type mice during airway infection with P. aeruginosa (32). At the molecular level, the flagellin of P. aeruginosa interacts with the extracellular domain of MUC1. During P. aeruginosa infection, the epidermal growth factor receptor (EGFR) associates with MUC1 and phosphorylates the MUC1 cytoplasmic tail. This phosphorylation induces interaction between MUC1 and TLR5 (33). In another study, it was shown that the immunosuppressive effect of MUC1 on TLR5 in bronchial epithelial cells is mediated by prevention of recruitment of MyD88 to TLR5 and inhibition of downstream signaling (34). A recent study reported that the extracellular domain of MUC1 can act as a releasable decoy molecule during P. aeruginosa infection, thereby restricting pathogen adherence to the epithelial surface (35).

H. influenzae is another important bacterial pathogen which can cause severe pneumonia and meningitis and other invasive infections such as septicemia, which particularly affects young children. During infection with nontypeable H. influenzae, bacterial lysates induce inflammation mainly through the activation of TLR2. Subsequently, TLR2 activation leads to production of TNF-α, which upregulates MUC1 through the activation of TNF receptor 1 (TNFR1), which in turn suppresses TLR2 signaling (36). MUC1 also inhibits signaling of other TLRs, including TLR2, TLR4, TLR7, and TLR9, during infection with respiratory syncytial virus (37). While TLR2 and TLR4 are localized at the plasma membrane where MUC1 is expressed also, TLR7 and TLR9 are located in the endosomal compartment. Which mechanisms explain the immunoregulatory effects of MUC1 on these different types of TLRs? First, the TLR extracellular domain could be shielded by the glycosylated extracellular domain of MUC1, which could subsequently reduce TLR-ligand interactions. Second, ligand binding and recognition could be hampered by the formation of a MUC1-TLR receptor complex, preventing direct interaction of pathogens with the epithelial cell surface. Third, the binding of MyD88 to the TLR intracellular tail could be impeded due to the direct interaction between MUC1 and TLR. Fourth, the cytoplasmic tail of MUC1 could act downstream by interacting with components of the NF-κB pathway and thereby modulate immune activation as was previously described for the role of MUC1 during Helicobacter pylori infection in the stomach (15).

S. aureus frequently colonizes the oral cavity and can cause respiratory disease in hospitalized patients and the elderly. One publication demonstrated that S. aureus associated with the secreted antimicrobial salivary mucin MUC7 during oral colonization (38). A recent study demonstrated strong binding affinity of a S. aureus surface adhesin regulated by the transcription factor MgrA to mucus in pig trachea. The surface adhesin and specific mucin glycoprotein involved in this interaction are yet to be characterized (39).

In addition to expression on epithelial cells, MUC1 is also expressed by immune cells, including macrophages. A recent study demonstrated that MUC1 facilitates phagocytosis of S. pneumoniae by macrophages and thereby limits bacterial infection. However, effects of MUC1 on phagocytosis seem to be highly bacterium specific. While mouse *Muc1* knockout macrophages exhibited a reduced capacity to phagocytose S. pneumoniae, phagocytosis of P. aeruginosa was increased compared to the level seen with wild-type macrophages (40). In addition to acting on phagocytosis at the plasma membrane, MUC1 is also an important suppressor of activation of the NLRP3 inflammasome. Stimulation of MUC1-deficient macrophages with either H. influenzae or S. aureus, either of which can activate NLRP3, resulted in elevated levels of proinflammatory IL-1β in comparison to wild-type macrophages (41). Overall, the existing data indicate that MUC1 plays an important anti-inflammatory role during infection with different bacterial pathogens at the respiratory mucosa.

In contrast to MUC1, information regarding the role of other transmembrane mucins (MUC4 and MUC16) in respiratory infections is still fragmentary. The role of these transmembrane mucins during airway infection needs to be addressed thoroughly to understand mucin-specific functions.

## ROLE OF MUCINS AND MUCIN GLYCANS DURING INFLUENZA VIRUS INFECTIONS

Influenza A virus (IAV) is a highly infectious respiratory pathogen that is a constant threat to the human population. IAVs are negative-sense, single-stranded, segmented RNA viruses and belong to the family Orthomyxoviridae. IAV can be categorized into subtypes based on the two surface proteins of the viral envelope: hemagglutinin (HA) and neuraminidase (NA) (42). Similarly to bacterial pathogens, IAV enters the respiratory tract through the nasal cavity and needs to traverse the soluble mucus layer and apical transmembrane mucins to be able to infect the underlying epithelial cells. Therefore, interactions with mucins and mucin glycans are essential steps in IAV pathogenesis. The protective role of mucins during IAV infection was demonstrated by overexpression of the soluble mucin Muc5ac in mice, which protected against viral infection (24, 43). This result is in line with the observation that upregulation of MUC5AC is part of the normal mucosal response during respiratory infections. A recent study showed increased morbidity and mortality in *Muc1* knockout mice infected with IAV compared to wild-type mice. Sialylated Muc1 directly interacted with viral particles and prevented IAV entry into respiratory epithelial cells. In addition, enhanced inflammatory responses were observed in *Muc1* knockout mice, which is in line with the anti-inflammatory effect of MUC1 during bacterial infection. McAuley et al. hypothesize that the anti-inflammatory activity of MUC1 could be mediated by the interaction of IAV with the extracellular domain of MUC1, release of the MUC1 extracellular domain, and shedding of the mucin-virus particle into the lumen (14).

Mucins of ciliated cells of the human upper respiratory tract primarily contain sialic acids that are bound to galactose by an α2,6 linkage, while α2,3-linked sialic acid predominates in the human lower respiratory tract on the bronchiolar nonciliated cuboidal cells and alveolar type II pneumocytes (44). In contrast, α2,3-linked sialic acids predominate on mucins in both the upper and lower respiratory tracts of avian hosts. Structural differences in sialic acid linkages play an important role in host restriction of the influenza virus. The human influenza viruses preferentially bind sialic acids with α2,6 linkages on extended glycans, while influenza viruses of avian and equine origin bind better to sialic acids with α2,3 linkages. The relative scarcity of α2,3-linked sialic acids in the upper respiratory tract of humans is thought to represent host restriction with respect to avian influenza viruses. The swine respiratory tract has been shown to express both α2,3-linked and α2,6-linked sialic acids, and for this reason, swine have been referred to as “mixing vessels” that foster reassortment of influenza viruses and increase the risk of zoonotic infections (45). Differential expression and activity of glycosyltransferases could lead to attachment of different sialic acid structures to mucins, but differences between species have not been characterized in detail.

## PATHOGENESIS OF RESPIRATORY CORONAVIRUSES

Coronaviruses are large, positive-sense enveloped RNA viruses in the Nidovirales order and are divided into four genera: α (e.g., human CoV-NL63 [HCoV-NL63] and HCoV-229E), β (e.g., HCoV-OC43 and HCoV-HKU1), γ, and δ. Several zoonotic β coronaviruses have caused several outbreaks of deadly pneumonia in humans since the beginning of the 21st century. The severe acute respiratory syndrome coronavirus (SARS-CoV) outbreak in 2002 was responsible for an epidemic with a fatality rate of 10% that spread to five continents. The Middle East respiratory syndrome coronavirus (MERS-CoV) emerged in the Arabian Peninsula in 2012 and has caused recurrent outbreaks in humans, with a fatality rate of 35%. The deadly coronavirus disease 2019 (COVID-19) pandemic caused by a novel coronavirus named SARS-CoV-2 started in late 2019. Coronaviruses infect cells by receptor-mediated entry using the envelope-anchored spike (S) protein that induces fusion of viral and host membranes. SARS-CoV and HCoV-NL63 use angiotensin-converting enzyme 2 (ACE2) as an entry receptor, whereas dipeptidyl peptidase 4 (DPP4) is recognized by the spike protein of MERS-CoV (46). One important feature of human coronaviruses is that, in addition to these entry receptors, they depend on sialic-acid-containing glycoproteins and gangliosides as primary attachment sites in the respiratory tract (47). In contrast, other coronaviruses such as HCoV-NL63, HCoV-229E, and porcine respiratory coronavirus (PRCoV) seem to lack sialic acid binding activity (48, 49).

Coronaviruses of low pathogenicity such as HCoV-OC43, HCoV-HKU1, bovine coronavirus (BCoV), and porcine hemagglutinating encephalomyelitis virus (PHEV) interact with 9-*O*-acetyl-sialic acid (9-*O*-Ac-Sia), a terminal residue corresponding to glycan chains on glycoproteins and lipids (50). Glycan array analysis revealed that MERS-CoV prefers to bind sulfated sialyl-Lewis^X^ with α2,3-linked sialic acid and, to a lesser extent, with α2,6-linked sialic acid. To further confirm the binding, lectin histochemistry analysis was performed in the upper and lower respiratory tissues of humans as well as of dromedary camels, the natural reservoir of MERS-CoV. The results demonstrated that α2,3-linked sialic acid is abundant in the camel nasal respiratory epithelium and the alveoli of the human lung. This specificity correlates with the predominant sites of MERS-CoV colonization in the upper and lower respiratory tracts of camels and humans, respectively (51). The study also provided evidence that neuraminidase treatment for removal of cell surface sialic acids inhibited MERS-CoV entry of Calu-3 human airway cells (52). This result suggests that interaction with surface sialic acids is essential for viral entry and indicates that sialic acids on soluble mucins could play a role in preventing MERS-CoV infection. Interestingly, the specific sialic acid binding site is present in MERS-CoV but in not found in SARS-CoV, which could possibly represent an important difference between the two viruses. However, an *in silico* study analyzed the interaction between the spike protein and its membrane receptors and, based on a quantitative characterization of surface regions of MERS-CoV spike and sialic acid molecules, the authors proposed that sialic acids may act as additional receptors of SARS-CoV-2 (53).

The roles of specific soluble and transmembrane mucins in coronavirus infection have not yet been studied in detail, but a study was recently deposited in bioRxiv that demonstrated that female *Muc4* knockout mice lost more weight than male *Muc4* knockout mice after infection with SARS-CoV and displayed increased disease severity (54). *Muc4* knockout mice had viral titers similar to those of wild-type (WT) mice but enhanced inflammatory cytokine responses. In addition, female *Muc4* knockout mice performed significantly more poorly than WT mice in pulmonary function measurements, including airway resistance and expiratory flow measurements. The results of that study suggest that the transmembrane mucin Muc4 plays an important protective role in female mice during coronavirus pathogenesis. The importance of Muc4 was also determined during pathology of the mosquito-borne Chikungunya virus, possibly indicating a broader importance of Muc4 during viral infection (54). Extensive research is needed to understand the role of transmembrane mucins and their glycans in coronavirus disease mechanisms.

## MUCOSAL DEFENSE AGAINST SARS-CoV-2

There is pressing urgency to understand the pathogenesis of COVID-19, which is mostly characterized by pneumonia, fever, cough, and occasional diarrhea. SARS-CoV-2 spike (S) protein binds ACE2 and, in concert with host proteases, principally TMPRSS2, promotes cellular entry (55). The clinical stages of COVID-19 can be divided into three phases based on the cells that are likely infected: (i) the asymptomatic state (the initial 1 to 2 days of infection); (ii) the upper airway and conducting airway response (the next few days); and (iii) hypoxia, ground glass opacity, and progression to acute respiratory distress syndrome (ARDS) (56). In stage 1, inhaled SARS-CoV-2 likely traverses the nasal mucus layer, invades nasal epithelial cells, and starts replicating. A recent single-cell RNA sequencing study generated by the Human Cell Atlas consortium reported the highest level of expression of ACE2 and TMPRSS2 in the nasal goblet and ciliated cells in healthy individuals (57). Single-cell transcriptome sequencing (RNA-seq) data analysis also revealed that the cluster of cells with ACE2 expression also expressed MUC1 (58) and the soluble mucin MUC5AC (59). These cells represent the most likely initial infection route for the virus, and nasal carriage is likely to be a key feature of transmission. The pore of the mucin gel is sufficiently large (approximately 500 nm) to ensure that it is readily penetrated by viruses which are generally 30 to 200 nm in diameter (4, 60). After initial replication, the virus propagates and migrates down the respiratory tract along the conducting airways, and a more robust infection is triggered. After acute infection, there may be less mucus production in the glands due to damage of glandular epithelial cells, leading to decreased mucus production and subsequent impaired mucociliary clearance. Subsequently, there are fewer droplets and virus particles and lower levels of other material cleared from the lungs, making the lungs more prone to infection (61). It is currently not known if and how SARS-CoV-2 interacts with human respiratory mucin and mucin glycans. However, a recent study indicated the presence of elevated levels of secreted MUC1 and MUC5AC in the sputum aspirated from the trachea of COVID-19 patients (62). COVID-19 patients also have a lower allelic frequency of the MUC5B genetic variant rs35705950 than healthy controls, which is associated with a higher level of expression of MUC5B, suggesting a protective role for MUC5B (63). The role of soluble and transmembrane mucins and their glycans during COVID-19 pathogenicity needs to be addressed to fully understand the course of the disease (Fig. 1C and D).

## IMPACT OF GENDER AND AGE ON MUCIN EXPRESSION—IMPLICATIONS FOR COVID-19

The elderly are at higher risk of SARS-CoV-2 infections that result in severe complications, with mortality higher in males than in females (64). Could it be that the gender and age differences that occur during respiratory diseases can be traced back to differences in mucin expression and glycosylation? In the final paragraph, we discuss changes to mucin expression, glycosylation, and lung function that relate to age and sex.

During cystic fibrosis, asthma, and chronic obstructive pulmonary disease (COPD), female patients generally have more-severe symptoms, poorer quality of life, and worse prognoses than male patients (65). Interestingly, it was previously demonstrated that expression and glycosylation of MUC5B and MUC5AC are regulated by the female sex hormone estradiol. In the human airways, the presence of estradiol increases the number of goblet cells, boosts expression of MUC5AC, and increases levels of expression of fucosyltransferases FUT4, FUT5, and FUT6 and total fucose residues (66). Due to the increased hydrophobicity of fucose residues, this increased fucosylation may negatively impact disease development in women suffering from chronic airway diseases (67). Estrogens also upregulate antibody sialylation, which yields an anti-inflammatory effect that is lost after menopause (68). Those authors speculate that reduction of sialylation of defensive respiratory mucus could contribute to the higher incidence of severe SARS-Cov-2 infections in men and elderly women (69). It has also been reported that interaction of 17β-estradiol with estrogen receptor alpha (ER-α) upregulates MUC5B gene expression through activation of the ERK-mitogen-activated protein kinase (MAPK) pathway (70). As mentioned above, female but not male *Muc4* knockout mice display increased disease severity after infection with SARS-CoV, suggesting that the Muc4 transmembrane mucin plays an important protective role in female mice during coronavirus pathogenesis (53). Furthermore, epidemiological data from both SARS-CoV infection and SARS-CoV-2 infection indicate more-severe disease in male mice, with the female mice acquiring their resistance at least in part from estrogen signaling, which in turn upregulates mucin gene expression (54, 71). Future studies into the expression of ACE2 in different tissues and its regulation by 17β-estradiol will help us understand the mechanisms that underlie these epidemiological findings (72, 73).

Due to aging of the human population, there is an increased incidence in pulmonary infections, associated with significant morbidity and mortality (74). The frequent occurrence of respiratory infections in the elderly could also be explained in terms of mucin. Decreased mucus production is a characteristic of aged lungs which can lead to weakened pathogen removal and increased susceptibility to respiratory infection and disease progression (75). Aging is also associated with lower mucociliary clearance due to changes in mucus property and a lower tracheal mucus velocity (76). Interestingly, old mice have a decrease in Muc5b mucin production and as a result a reduction in mucociliary clearance in comparison to young mice (77). On the basis of the studies cited here, the higher susceptibility of elderly individuals to SARS-CoV-2 may in part be explained by decreased mucus production and impaired MCC allowing the virus to descend to the alveoli more readily in aged individuals. Furthermore, it was recently demonstrated that lower sialic acid content of the respiratory mucosa associated with aging leads to more-favorable conditions for initiation of viral infections (69). These findings make the research area of age-related and gender-related changes to respiratory mucins and mucin glycans an important field for future studies.

## CONCLUSION

The mucosal barrier is the body’s first line of defense in fighting infection, and it offers protection from noxious pathogens. Mucins, the major structural components of mucus, are complex glycoproteins. In addition to acting as a physical barrier, they are key players in cell signaling, inflammation, and carcinogenesis. Understanding specific interactions of bacterial and viral pathogens, including SARS-CoV-2, with respiratory mucins could aid in the development of tailor-made drugs that block these interactions and prevent pathogen invasion. Another promising strategy could be to upregulate mucin expression or alter glycosylation in order to boost mucosal defense mechanisms and prevent respiratory infections.
